# Hot-Rolling and a Subsequent Direct-Quenching Process Enable Superior High-Cycle Fatigue Resistance in Ultra-High Strength Low Alloy Steels

**DOI:** 10.3390/ma13204651

**Published:** 2020-10-18

**Authors:** Min-Seok Baek, Young-Kyun Kim, Tae-Won Park, Jinhee Ham, Kee-Ahn Lee

**Affiliations:** 1 Department of Materials Science and Engineering, Inha University, Incheon 22212, Korea; msbaek7@inha.edu (M.-S.B.); youngkyun@inha.edu (Y.-K.K.); 2 Agency for Defense Development, Daejeon 34186, Korea; parktw9@add.re.kr (T.-W.P.); jhham@add.re.kr (J.H.)

**Keywords:** direct quenching process, ultra-high strength steel, hot-rolling reduction rate, tensile, high-cycle fatigue, deformation behavior

## Abstract

The current study investigated the effect of hot rolling reduction rate of ultra-high strength low alloy steel manufactured via the direct quenching process on microstructure, tensile and high-cycle fatigue properties of the alloy. In order to control the reduction rate of ultra-high strength steels (UHSSs) differently, the steels were produced with two different thicknesses, 6 mm (46.2%—reduction rate, A) and 15 mm (11.5%—reduction rate, B). Then, the two alloys were directly quenched under the same conditions. Both the UHSSs showed martensite in the near surface region and auto-tempered martensite and bainite in the center region. Tensile results showed that alloy A with higher fraction of finer martensite had higher yield strength by about 180 MPa (1523 MPa) than alloy B. The alloy A was also found to possess a higher tensile strength (~2.1 GPa) than alloy B. In addition, alloy A had higher strength than B, and the elongation of A was about 4% higher than that of alloy B. High-cycle fatigue results showed that the fatigue limits of alloys A and B were 1125 MPa and 1025 MPa, respectively. This means that alloy A is excellent not only in strength but also high-cycle fatigue resistance. Based on the above results, the correlation between the microstructure and deformation behaviors were also discussed.

## 1. Introduction

With the advancements in the automobile and aviation industries, the demand for extremely high performance steels is also rising. Diverse new steel materials are being developed to meet such requirements, and out of these materials, the ultra-high-strength steel (UHSS) is expected to be a useful structural material in the aviation and automobile industry due to its high strength-ductility, superior toughness, and appropriate formability [1,2,3,4,5,6]. Currently, the strength of the commercially available UHSS is about ~1.2 GPa. Great efforts are still continuing to develop steels with higher strength and toughness than the commercial ones [7,8,9].

Typically, UHSS, with bainite and tempered martensite structures, is generally processed via a quenching & tempering (Q&T) heat treatment [10]. In this process, depending upon the Q&T heat treatment conditions, the phase fracture varies and the desired strength and elongation can be properly controlled. However, to implement tempering, re-austenitizing (RA) should be performed, but again that costs a lot of time and money [11]. Diverse methods have been presented to overcome such weaknesses, and among them, the direct-quenching (DQ) gained extensive attention because it can remarkably reduce the procedural steps.

The DQ process is one of the thermo-mechanically controlled processes (TMCPs) and a novel and effective method to fabricate high-strength and high-performance steel. This process involves spraying of pressurized water immediately after hot forging or hot forming to produce boards [12,13,14,15]. In this process, during hot forming, recrystallization takes place in the austenite phases, which results in grain refinement. In addition, during the hot forming, dislocation density was also found to increase in the austenite phase. Such dislocation density and grain size changes in the hot forming process have an impact on the martensite fraction of the direct-quenched steel [8,9]. Thus, the steels produced via the DQ process have higher martensite fraction than the conventional re-austenitizing and quenching (RA/Q) steels., The steels produced via the DQ process have also been reported to possess excellent mechanical properties [16,17,18,19]. Steel plates show different levels of strain accumulation depending upon hot rolling reduction rate, which actually brings a large change in the microstructure of the final plate. Since the DQ plates have no conventional pre-austenitizing process, it is more important to change the hot rolling reduction rate initially to control the strain level. In the DQ process, the higher the initial reduction rate, the thinner and stronger the plates formed. However, at the same time, some defects might arise on the steel plate surface [8]. Generally, defects on the surface and surface area are due to degraded mechanical properties, particularly, the high-cycle fatigue property. Therefore, controlling the defects on the surface is essential for DQ UHSSs. Recently, several studies have been actively pursued to control the mechanical properties by optimizing the DQ process conditions [9,10,18]. In some studies, attempts were made to control the mechanical properties of conventional steels through microstructure evolutions, such as carbide formation and retained austenite stabilization by utilizing heat treatment [20]. In other words, the DQ process is thought to be a novel process through which mechanical properties can be controlled only by changing the process conditions without any additional heat treatment.

For directly quenched steel plates, pressurized water is sprayed on the plate surface so that difference in cooling rate lies in each part of the near surface region (NSR) and center region (CR). Such difference in the cooling rate causes auto-tempering inside the plates, which possibly leads to the formation of both martensite and bainite simultaneously. The bainite formation via auto-tempering can induce a combination of excellent strength and impact toughness. Thus, the cooling rate control becomes significant factor in the DQ process [14,21]. If the initial hot rolling reduction rate is different, the thickness of the steel plate can be different, and thus, the cooling rate applied to the material during the DQ process is also changed.

Tensile properties and fracture toughness of direct-quenched steels have already been reported in the past [15,16,17,18,19,20,21]. Considering their superior properties of tensile and fracture toughness, DQ steels can be used in automobile and vessel parts, armored vehicle shells, and construction materials. However, prior art reported just the simple mechanical properties and not any profound details on the correlation between the microstructure and mechanical properties. More specifically, to use UHSS as a practical structural material, it is essential to understand the high-cycle fatigue properties and the effects of microstructural factors on fatigue deformation behavior. However, no studies have been reported to-date on this topic.

The present study has investigated the effect of hot-rolling and subsequent direct-quenching on the microstructure, tensile and high-cycle fatigue properties of UHSS. In addition, the effects of the microstructure features changed by the reduction rate and cooling rate difference on the tensile and high-cycle fatigue deformation behavior were also examined.

## 2. Experimental Procedure

**Figure 1:**
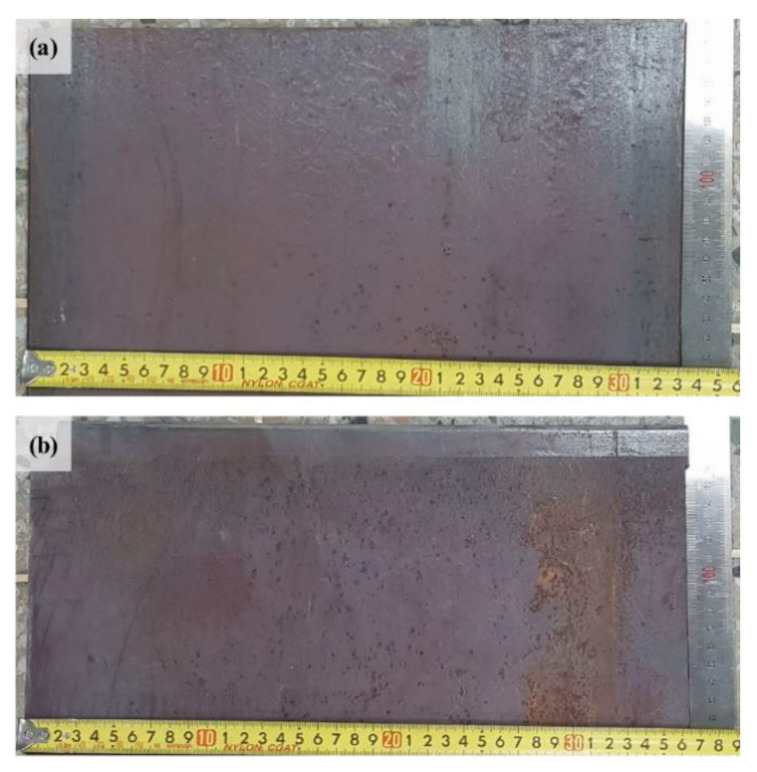
Surface morphology of steel plates after direct quenching process: (**a**) alloy A (5T) and (**b**) alloy B (15T).

Table 1 lists the composition of the steel samples studied in the present work. Inductively coupled plasma (ICP) analysis found that the fabricated ultra-high strength steel contained 0.3 wt. % or lower C along with Ni, Cr, Mo, and Mn. A 130 mm-thick steel slab was reheated at 1170 °C for 3 h. Then, hot rolling was performed with two types of plates, one of thickness 6 mm (alloy A) and the other of thickness 12 mm (alloy B). The final rolling temperature was set at 850–870 °C. This temperature range is lower than the recrystallization temperature (T_NR_) as defined by the Boratto equation [22]. As reported already, if hot rolling is implemented at lower temperature than T_NR_, the shape of an austenite grain becomes elongated (pancaked austenite) along the rolling direction [14]. After the hot rolling, pressurized water was utilized for direct quenching to room temperature. Figure 1 shows photographs of the two types of UHSS plates produced via the above process. Both the materials showed no cracks on the surface of the plates and were found to have been soundly manufactured.

To analyze the phases and microstructures of the two UHSSs, viz. alloy A and B, the prepared specimens were polished with a polisher up to #100~#1200 (sandpaper grit). They were also mirror-polished together with 3 μm and 1 μm diamond pastes using 0.01μm colloidal silica. For phase analysis, X-ray diffraction (XRD, X’Pert-PRO MRD, Malvern Panalytical, Malvern, UK) was used at a scan speed of 4°/min and 2-theta (θ) 20~100°. To observe the microstructural difference between the alloys A and B, field-emission scanning electron microscope (FE-SEM, MYRA 3, TESCAN, Brno, Czech), and high-resolution electron back-scattered diffraction (HR-EBSD, OXFORD, Abingdon-on-Thames, UK) studies were conducted. The HR-EBSD analysis conditions were–tilting angle: 70 degrees, acceleration voltage: 20 kV, beam intensity: 13, and step size: 50 nm. Field-emission transmission electron microscopy (FE-TEM, JEM-2100F, JEOL, Akishima, Japan) micrographs were also taken. The TEM specimen was prepared using jet-polisher (TenuPol-5facts, STRUERS; Copenhagen, Denmark) in glacial acetic acid solution at 15 V and 250 mA. 

To obtain the mechanical properties, tensile tests were carried out for both the UHSSs using 8501 equipment (INSTRON, Norwood, USA). The tensile specimen was processed following the ASTM E8 M standard and 10^−3^ s^−1^ strain rate was used in the test. Each material was tested 5 times and the average value was used for the analysis.

High cycle fatigue test was done using INSTRON 8501 following the ASTM E466 standard. The conditions set for the fatigue test are as follows. The stress ratio (R) was 0.1 while the frequency was 20 Hz along with sinusoidal stress control. The fatigue specimen’s gage part was first polished and then tested 3 times for each cyclic stress condition. The FE-SEM analysis was utilized for tensile, fatigue fracture surface and cross-sectional observations.

## 3. Results

### 3.1. Microstructure and Phase Analysis Results of the Directly Quenched UHSSs

**Figure 2:**
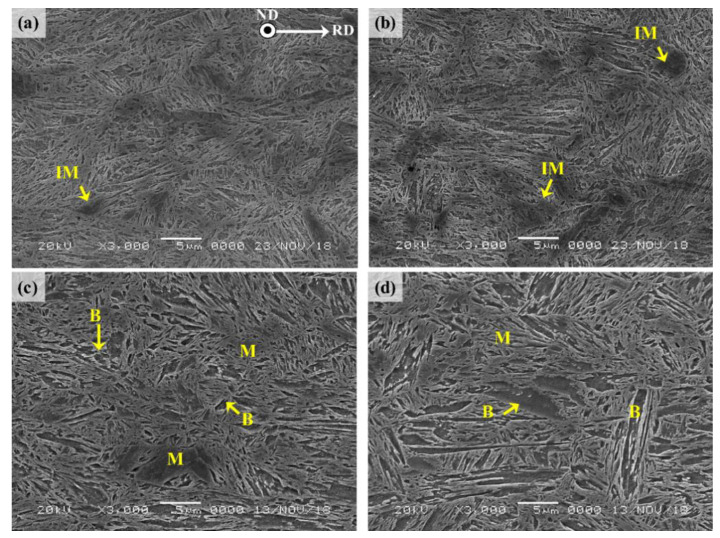
Scanning electron microscope images of the initial microstructures of near surface region: (**a**) alloy A; (**b**) alloy B, and center region: (**c**) alloy A and (**d**) alloy B.

For the UHSSs produced via the DQ process, the microstructure of each material generally varies according to the rolling and heat treatment conditions (final rolling temperature (FRT)). For this reason, the NSR and CR were separated to observe the microstructures, and the results are shown in Figure 2. The NSR observation results indicated that the two steel samples consisted of martensite structure for the most area (Figure 2a,b). Some island martensites (IMs) were also observed, and their sizes were similar in both the UHSSs. On the other hand, the center region (CR) was found to have bainite structure in addition to martensite in the two alloys (Figure 2c,d). It is already reported that a temperature gradient typically exists from the surface to the core in the DQ process and this induces auto-tempering in CR [12,13,14,15,23,24]. The bainite fractions in the CR of the alloys A and B were measured to be 14.71 vol. % and 27.18 vol. %, respectively, demonstrating a larger amount of bainite formation in the alloy B.

**Figure 3:**
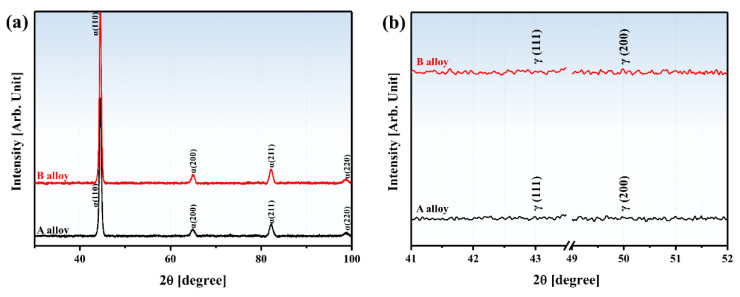
(**a**) XRD spectra of direct-quenched steels: alloy A (black line) and alloy B (red line); (**b**) XRD intensities of γ (43.28° (111) and 50.40° (220)) peaks.

Figure 3 shows the X-ray diffraction analysis results of alloys A and B. Both the alloys showed only α’-peaks which could be martensite or bainite (Figure 3a). However, no austenite (γ) phase was found, which is generally formed in low carbon steel (Figure 3b). This is because low carbon steel has γ phase mostly in the lath martensite interface and its thickness is approximately ~120 nm [25,26]. Since the γ phase is considerably thin, as a result, it is difficult to detect in XRD results.

**Figure 4:**
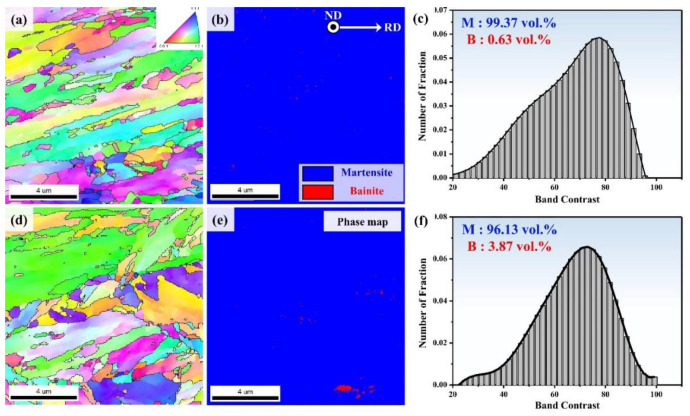
(**a**) IPF map of near surface region of direct-quenched steel (alloy A) (**b**) with corresponding selected area phase map and (**c**) Gaussian graphs in (**a**) revealing prominent martensite (blue) & bainite (red); (**d**) IPF map of alloy B (**e**) with phase map and (**f**) Gaussian graphs in (**c**).

Figure 4 shows the EBSD analysis results (band contrast (BC), inverse pole figure (IPF) and phase map) at the NSR of the two UHSSs. In the IPF maps (Figure 4a,d), prior austenite grain (PAG) size of A and B alloys were measured as 10.37 μm (±1.13 μm) and 11.71 μm (±1.18 μm), respectively. This means that alloy A had slightly smaller PAG size. The phase map (Figure 4b,e) showed that both the alloys consisted of mainly martensitic structure in the NSR. Here, the martensite/bainite (M/B) fraction was quantified using EBSD band contrast (BC) map analysis. The BC-based M/B fraction analysis method involves measurement on the basis of kikuchi patterns, i.e., the diffraction pattern intensities of martensite and bainite [27,28]. In general, martensite has greater lattice imperfections (carbon solute, dislocation, and low angle boundary) than bainite, resulting in a lower kikuchi pattern intensity in the former. Therefore, if EBSD beam scanning is carried out at the same time over the same area, the kikuchi pattern quality of martensite with greater lattice imperfections is lower (blurrier) than that of bainite. Such lower kikuchi pattern intensity of martensite leads to a lower band contrast value. The M/B fraction analysis revealed that the martensite fraction in alloy A was 99.37 vol. % and in alloy B was 96.13 vol. %, demonstrating that alloy A had a higher martensite fraction than alloy B.

**Figure 5:**
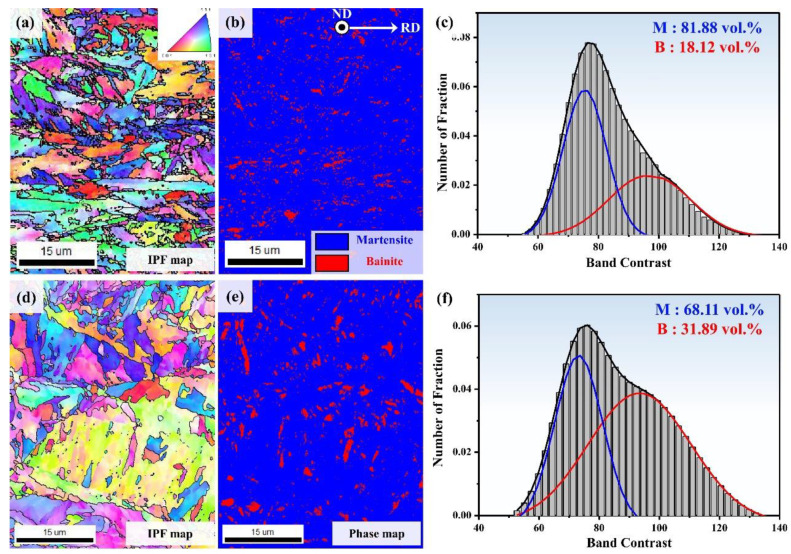
(**a**) IPF map of center region of direct-quenched steel (alloy A) (**b**) with corresponding selected area phase map and (**c**) Gaussian graphs in (**a**) revealing prominent martensite (blue) & bainite (red); (**d**) IPF map of alloy B (**e**) with phase map and (**f**) Gaussian graphs in (**c**).

Figure 5 presents the EBSD results for the CR. First, the IPF map (Figure 5a,d) shows that alloy A had more elongated PAG along the rolling direction than alloy B. The PAG size was measured to be 21.73 μm (±3.31 μm) for alloy A and 34.17 μm (±6.79 μm) for alloy B. In the NSR, the PAG sizes were similar for both the alloys. However, in the CR, alloy A had significantly smaller PAG size than alloy B. This is because the strain accumulation in the CR during rolling was relatively higher in alloy A than in B. The CR phase map analysis showed that the martensite fraction in alloy A was 81.88 vol. % and in alloy B was 68.11 vol. %, whereas the bainite fractions were 18.12 vol. % in A and 31.89 vol. % in B. As mentioned above, the steels manufactured through the DQ process were reported to possibly have auto-tempered structure [23,24]. In other words, although the board NSR mainly had martensite structure due to fast cooling rate, the bainite structure could be additionally created in the CR due to auto-tempering. Moreover, if the plate thickness is relatively thicker as for alloy B, the fraction of the bainite structure would be found higher. The difference between the two materials’ fraction was because of varying auto-tempering effect due to initial deformation difference by hot rolling reduction rate and different cooling rate due to thickness change.

**Figure 6:**
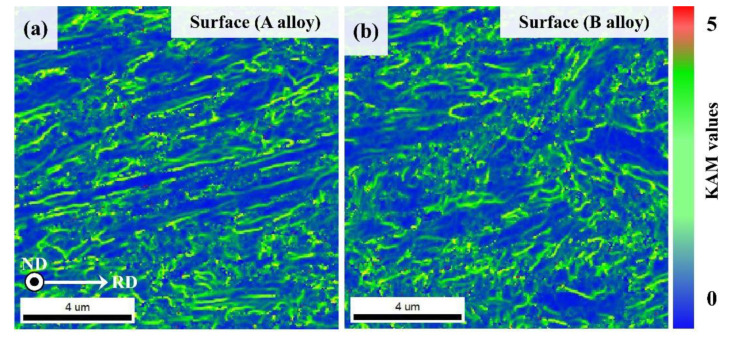

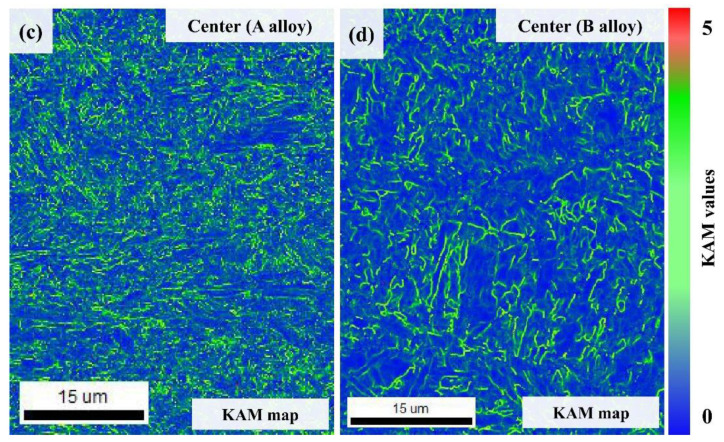
KAM maps of the initial microstructures of near surface regions of directly quenched steels (**a**) alloy A, (**b**) alloy B, and center region; (**c**) alloy A and (**d**) alloy B.

Figure 6 shows the results of kernel average misorientation (KAM) map analysis of the two alloys in their NSR and CR. In the KAM maps, the KAM values concentrated on the martensite block & lath boundary (near surface region), and martensite and bainite block boundary (center region) of both the UHSSs steels. The two UHSSs’ KAM values showed similar distributions in the NSR, whereas, in the CR, alloy A’s KAM distribution was more uniform than that of alloy B. This means that alloy A had relatively finer PAG and martensite packet than alloy B.

**Figure 7:**
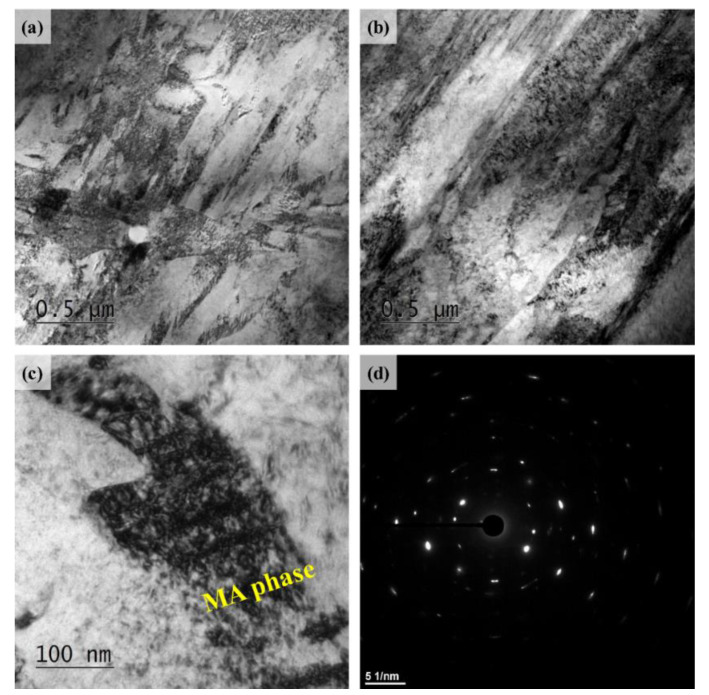
Bright field TEM images of the lath structure (tempered martensite & bainite) of center regions of the direct-quenched steels (**a**) alloy A, (**b**) alloy B; (**c**) bright TEM image of the martensite-austenite (MA) phase (**d**) corresponding selected area diffraction pattern (SAED) of (**c**).

Figure 7 shows the TEM micrographs of the plate type auto-tempered-martensite and bainite of the two UHSSs. 

Since the SEM and EBSD results already showed that the two alloys had similar microstructures in the NSR, TEM studies were carried out only for the CR where distinct microstructural differences were observed. The TEM images indicated that alloy A, compared to alloy B, had finer martensite and bainite phases. Moreover, alloy B showed an additional martensite-austenite (MA) phase which was not observed in alloy A. Generally, when steel has a low cooling rate, the carbon diffuses rapidly inside the austenite to make excessive flocculation in some region(s) [29,30]. Some austenite which have excessive carbon content can hardly induce diffusionless martensite transformation, which leads to the formation of an MA phase in this region [29,30,31,32]. 

**Figure 8:**
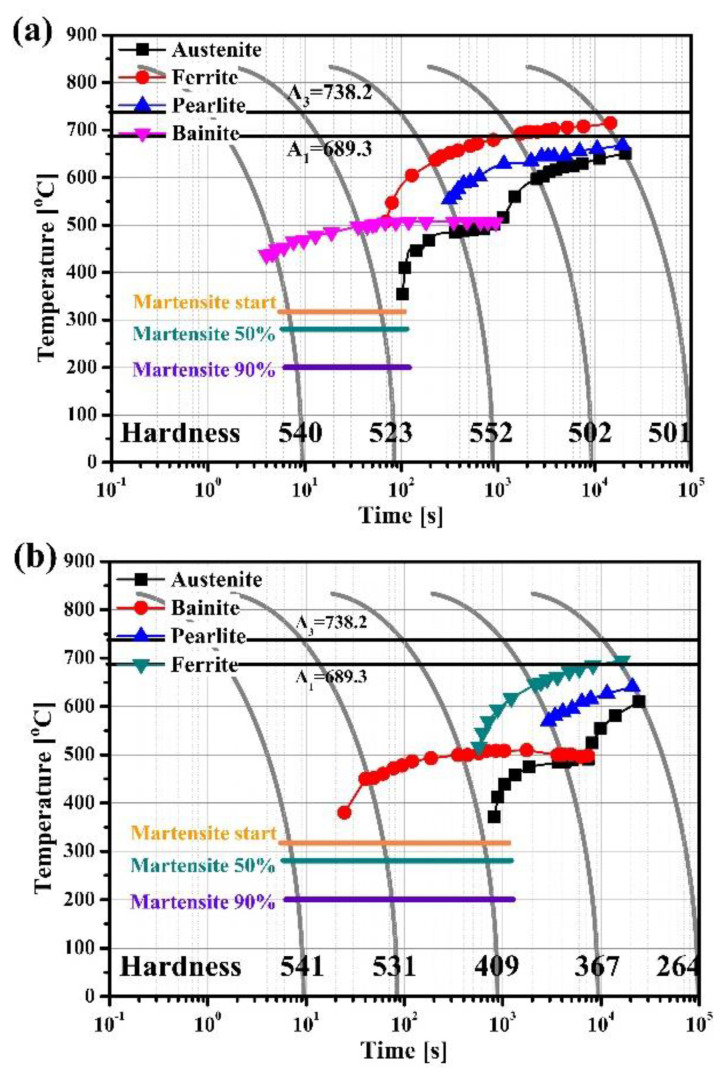
Simulated continuous cooling transformation (CCT) results using JMatPro program for: (**a**) alloy A and (**b**) alloy B.

Since rapid cooling starts from the surface, temperature gradient exists inside the involved material, and the CR has a relatively slower cooling rate. In this regard, the continuous cooling transformation (CCT) curves of the two alloys’ CR were calculated using JMatPro (Sente Software Ltd., Guildford, UK) (Figure 8). The total time (s) for cooling completion of the alloys A and B were 20 s and 180 s, respectively. These results quantitatively demonstrated that alloy B had a far lower cooling rate than alloy A. Therefore, if hot rolling reduction rate is low (alloy B, thicker plate), a material’s cooling rate in the CR is considerably low and it is possible to form the MA phase.

### 3.2. Mechanical Properties and Deformation Behaviors of Directly Quenched UHSSs 

**Figure 9:**
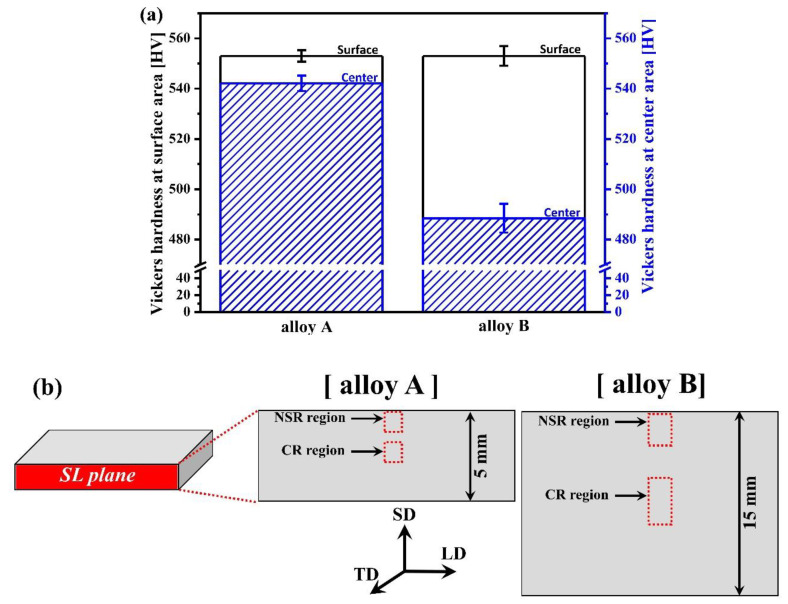
(**a**) Comparison of Vickers hardness of near surface & center region of direct-quenched steels (alloys A and B); (**b**) Vickers hardness measured area of alloys A and B.

Figure 9a shows the results of the two alloys’ Vickers hardness. For hardness test, the NSR and CR were separately measured and compared. The Vickers hardness was checked cross-sectional (SL plane) of the DQ steels, and the measured areas are shown in Figure 9b. Total 20 hardness measurements were performed for each area, and the average value excluding the largest and lowest values was used. The NSR hardness of the two alloys was the same, i.e., 553 HV. The hardness deviation found for the two alloys was small, ±2.3 (alloy A) and ±3.9 (alloy B). The hardness values of the CR were 542 HV (±3.1) for alloy A and 488 HV (±5.7) for alloy B, indicating that alloy B had a far lower value than alloy A. It was found that alloy A had a relatively uniform hardness distribution between NSR and CR compared to alloy B.

**Figure 10:**
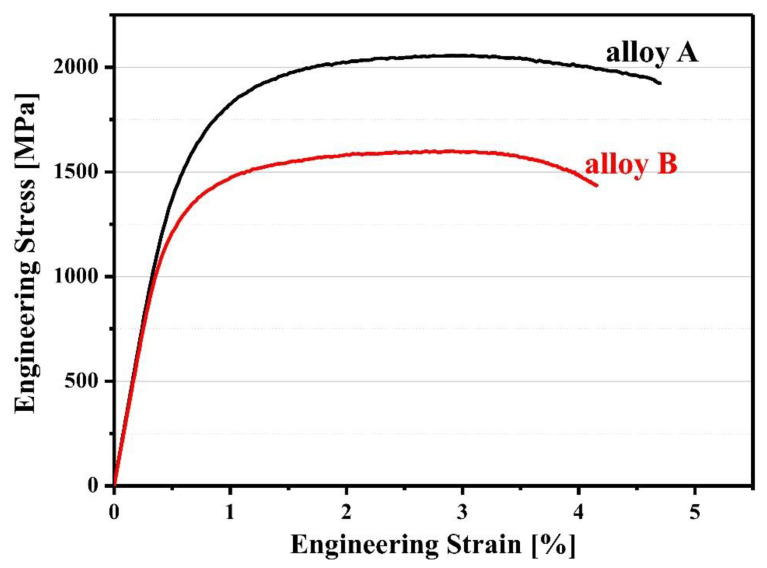
Comparison of tensile curves of direct-quenched steels (alloys A and B)

The tensile stress-strain curves of the directly quenched UHSSs have been depicted in Figure 10, and the tensile results have been summarized in Table 2. Yield strength was measured by using 0.2% offset method in the obtained engineering stress vs. engineering strain curve. The alloy A had higher yield strength and tensile strength than the alloy B. The alloy A showed tensile strength of around ~2.1 GPa, which is an exceptional tensile property among the low-alloy steels. This is because alloy A had finer martensite size (packet & block) than alloy B and had a high fraction of martensite. Furthermore, the true strain of alloy A was 0.042 and that of alloy B was 0.043, showing similar elongation regardless of their strength difference. As DQ or TMCP-processed steels have a very high yield strength, their properties can be compared using yield- and tensile-strength ratio (YTR) which indicates the degree of work hardening, other than the common engineering strain [5,33,34]. The two alloys’ calculated YTR values were 0.725 for alloy A and 0.728 for alloy B. To note, 1 GPa-level TMCP steels have the YTR value of about 0.75 [35,36]. Also, as the strength increases, YTR increases but the work hardening does not occur significantly. The present UHSSs with tensile strength of ~2.1 GPa have YTR lower than 0.75, indicating the possibility of greater work hardening.

### 3.3. High Cycle Fatigue Properties and Fatigue Behaviors of Direct-Quenched UHSSs

**Figure 11:**
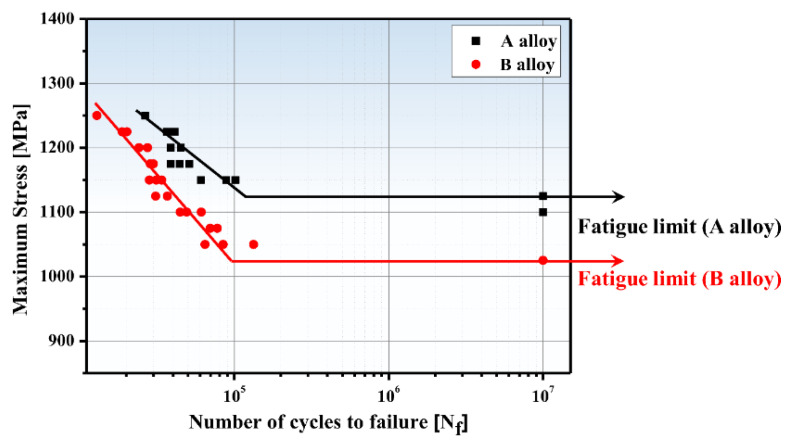
Comparison of high-cycle fatigue properties (S–N curves) of alloys A and B.

Figure 11 presents the S-N (cyclic stress (S) and cycles to failure (N_f_)) curves obtained from the high-cycle fatigue tests of the two UHSSs at room temperature. The fatigue limit (condition of 10^7^ cycles to failure) of alloy A was 1125 MPa and that of alloy B was 1025 MPa. Under all the fatigue stress conditions, alloy A showed an excellent fatigue life. 

Generally, it is known that materials with stronger tensile strength have better properties. This was also found true in the present study. To find the fatigue strength exponent (*b*) value in the S-N curves, Basquin’s law [37,38] was applied:

(1) $$\sigma_{a}={\sigma^{\prime }}_{f}{\left(2N_{F}\right)}^{b}$$

In the equation, *σ_a_* is stress amplitude, *N_f_* the number of cycles to failure, ${\sigma^{\prime }}_{f}$ the fatigue strength coefficient, and *b* the fatigue strength exponent. The *b* values for alloys A and B are 0.06, 0.09, respectively, which signified a smaller absolute value for alloy A. The smaller absolute *b* value means that the growth value of fatigue life (cycles (N_f_)) increases more as the maximum stress decreases.

## 4. Discussion

### 4.1. Tensile Deformation Behaviors 

**Figure 12:**
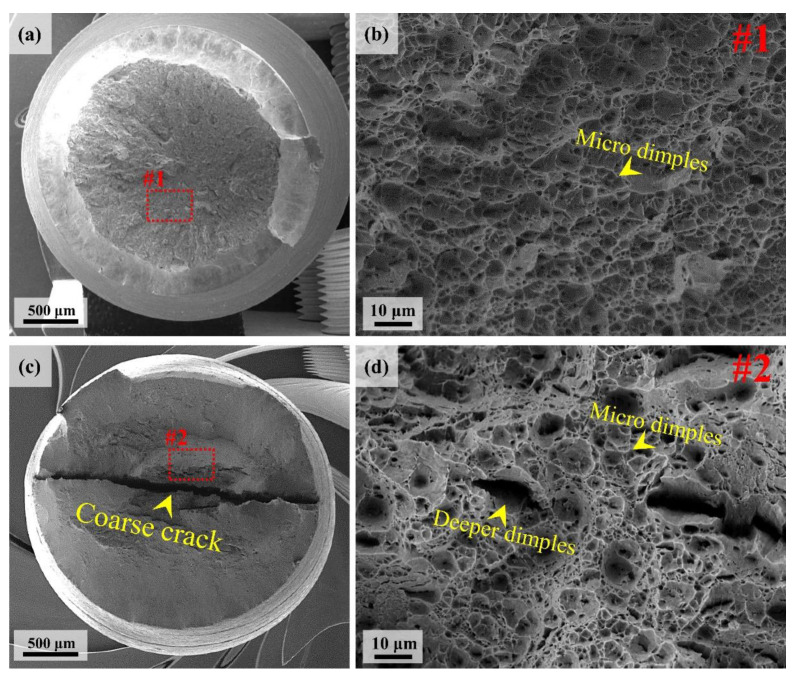
(**a**) SEM overview image of tensile fractography of alloy A; (**b**) high magnification SEM image of microscale dimples; (**c**) SEM overview image of tensile fractography of alloy B, and (**d**) high magnification SEM image of micro & deeper dimples.

The tensile fracture surface of the UHSSs was studied and the results are shown in Figure 12. While alloy B had coarse cracks in the central part of the fractured surface, alloy A had no such coarse cracks. Moreover, alloy B had a lot of secondary cracks in addition to the coarse cracks. The fractured surface was observed under high magnification. As a result, both the alloys had dimples, the typical characteristic of ductile fracture mode. Furthermore, alloy A had same-sized dimples, whereas alloy B had two types of dimples, the micro dimple and deeper dimple. The deeper dimples were reported to be commonly observed in bainitic steel [39,40]. It is also known that if the bainite size is coarse, stress and deformation concentrate at the bainite boundary to form cracks easily [39,40,41]. The alloy B had a higher bainite fraction to create deeper dimples and secondary cracks. According to Li et al. [29], MA phase is generated in PAGB when cooling rate is low. Such MA phase works as crack initiation site and has negative effect on the mechanical properties [29,30,31]. The present study found that alloy B had a large amount of MA phase in the CR due to the low cooling rate (Figure 7c). Considering the findings above, it seems that the presence of coarse cracks in alloy B was because MA phase was intensively created in the CR and its fraction was high in alloy B (Figure 12d).

**Figure 13:**
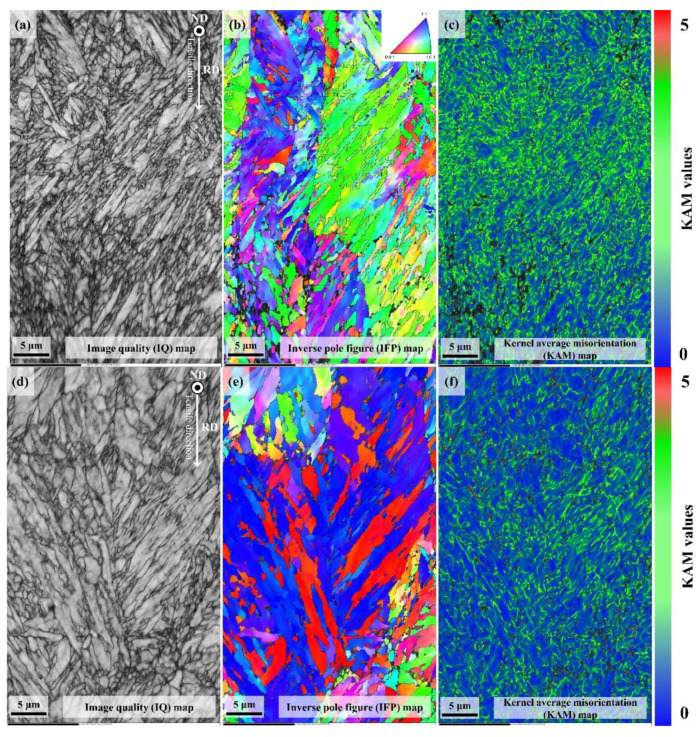
(**a**) IQ map of tensile deformed microstructure; (**b**) IPF map and (**c**) KAM map of alloy A; (**d**) IQ map; (**e**) IPF map and (**f**) KAM map of alloy B.

Figure 13 shows the EBSD IPF maps and KAM maps of the cross-sections of the areas immediately beneath the tensile fracture surface of alloys A and B. From the IQ and IPF maps, similar to the microstructure of the as-quenched, alloy A had finer PAG and martensite packet sizes than alloy B. Marinelli et al. [41] mentioned that the M/B interface or martensite block boundary acts as a strengthening barrier for effective dislocation motion. In other words, the finer the martensite structure, the more the strengthening barrier sites are. Therefore, alloy A should have better strength than alloy B. Moreover, the KAM distribution analysis revealed that after tensile deformation, both the alloys had high angle boundaries concentrated on strain, which was the M/B or martensite block interfaces. The KAM distribution results also showed that the strain distribution was more even after deformation in alloy A than that in B. On the other hand, alloy B showed coarse KAM distribution after deformation, indicating relatively less homogeneity. The M/B interface worked as a strengthening barrier and stress concentration site. Such stress concentration can cause cracks to undermine the mechanical properties [39,40,41]. Alloy B had coarse martensite structure and inhomogeneous strain distribution, resulting in easier crack formation than alloy A. For this reason, alloy B showed similar elongation as alloy A although the former’s strength (Y.S and U.T.S) was lower than the latter.

### 4.2. High-Cycle Fatigue Behaviors

**Figure 14:**
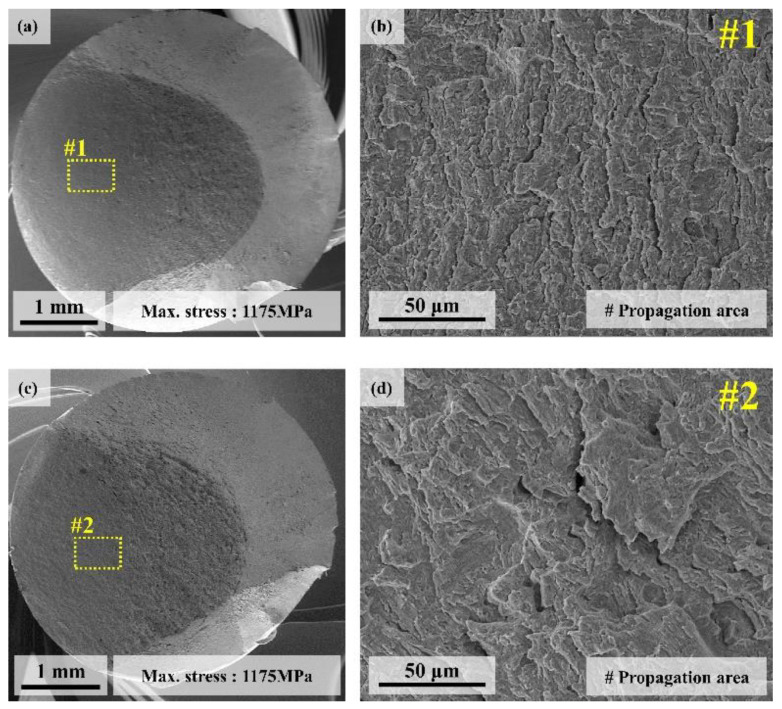
(**a**) SEM overview image of high-cycle fatigue fractography of alloy A; (**b**) high magnification SEM image of fatigue crack propagation area; (**c**) SEM overview image of high-cycle fatigue fractography of alloy B, and (**d**) high magnification SEM image fatigue crack propagation area.

Figure 14 exhibits the high-cycle fatigue fracture surfaces of the two UHSSs. None of the alloys showed any defects such as inclusion in the fatigue initiation region. 

**Figure 15:**
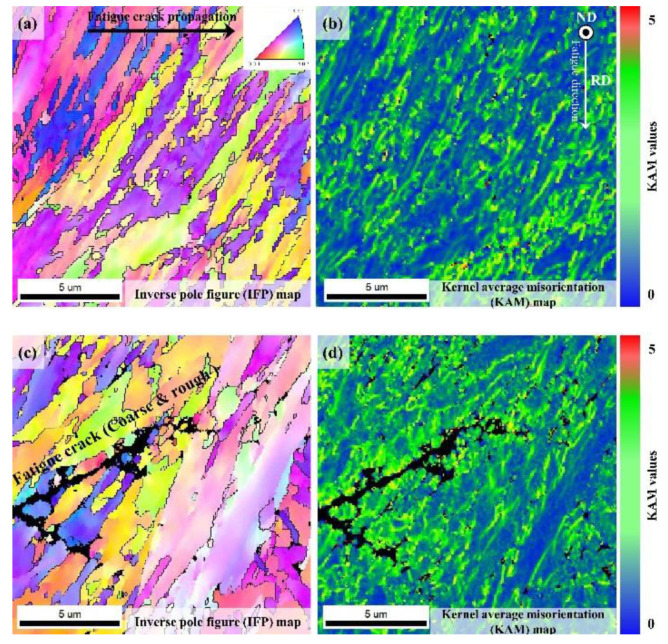
(**a**) IQ map observed after high-cycle fatigue; (**b**) KAM map of alloy A; (**c**) IQ map and (**d**) KAM map of alloy B.

Close observation of the fatigue propagation region revealed that alloy A had secondary cracks like fatigue crack tip. Similar fatigue cracks in the fatigue propagation region was also observed for alloy B, but unlike alloy A, those cracks were rough and slightly coarser. In general, high cycle fatigue life is closely related to crack initiation. Therefore, the cross-section in the region immediately beneath the fatigue crack initiation was analyzed using EBSD and the results have been displayed in Figure 15. First, alloy A did not show any secondary crack near the crack initiation region (Figure 15a,b).

By contrast, alloy B showed rough and slightly coarse secondary cracks in the region directly under crack initiation which were also observed in the fatigue propagation region (Figure 15c,d). The slightly coarse cracks of alloy B were created at the martensite block boundaries. The two UHSSs’ EBSD KAM map showed that the distribution of average KAM values was concentrated at the M/B and martensite block boundaries. In the high-cycle fatigue under repeated load, the M/B and martensite block boundaries can work as stress concentration sites [42,43,44]. In other words, if cyclic loading was applied under yield strength, the M/B and martensite block boundaries could be weaker than the MA phase, unlike in the case of uniaxial tensile test.

**Figure 16:**
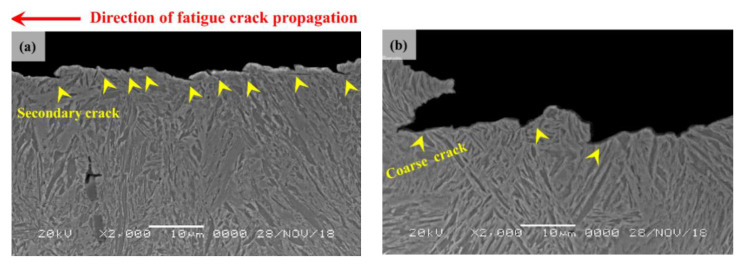
(**a**) Cross-sectional SEM image showing secondary cracks in the fatigue crack propagation area in alloy A, and (**b**) cross-sectional SEM image showing coarse cracks among PAGB in the fatigue crack propagation area in alloy B.

Figure 16 exhibits the two UHSSs’ cross-sectional view of the fatigue crack propagation region. Alloy A displayed many fine secondary cracks. When secondary cracks are formed, crack propagation paths are generally split to delay fatigue fracture [45]. The alloy B, on the other hand, showed relatively coarse and rough cracks, which, as reported earlier, weaken the fatigue properties [45]. A closer look at the cross-section of alloy B revealed that the coarse cracks were of similar forms and sizes to those of the PAG. In the initial microstructure studies (see Figure 7), creation of the MA phase was mentioned in the PAGB of alloy B. The findings indicated that although the MA phases did not work as crack initiation site in cyclic loading atmosphere at yielding strength or lower, it can be presumed to facilitate fatigue fracture in the fatigue propagation region.

## 5. Conclusions

The present study investigated the effects of hot rolling reduction rate on microstructure, tensile and high-cycle fatigue properties of ultra-high strength steel (UHSS) processed via the direct-quenching (DQ) method. The important conclusions derived are as follows: 

(1) In order to control the UHSSs’ reduction rate differently, the material thickness was set to 6 mm for alloy A and 15 mm for alloy B. EBSD results showed that martensite structure was primarily present in both the alloys’ near surface region (NSR), and their prior austenite grain (PAG) sizes were similar. In the center region (CR) of the two alloys, tempered martensite and bainite were observed, and alloy A had a higher martensite fraction. The PAG sizes in the CR were finer in the alloy A than in alloy B. TEM images of the CR showed that alloy B had martensite-austenite (MA) phases which did not form in alloy A. This was because alloy B, compared to alloy A, was thicker due to a lower hot rolling reduction rate, and the cooling rate inside the material was relatively lower. 

(2) The tensile test results showed that alloy A had YS: 1523 MPa and UTS: 2100 MPa, while alloy B had YS: 1373 MPa and UTS: 1883 MPa, indicating excellent strength of alloy A. Elongation showed similar values—total El.: 4.2% (alloy A) and 4.3% (alloy B). Tensile fracture surface analysis found that alloy B, unlike alloy A, had coarse and many secondary cracks in the center of the fracture surface. This was attributed to the MA phases concentrated on the PAG in the center of alloy B and formation of coarse bainite.

(3) High-cycle fatigue test found that alloy A had better fatigue properties than alloy B under all stress conditions. This was because alloy A was made at high hot rolling reduction rate, and had high martensite fraction after DQ and fine PAG sizes. Fatigue fracture surface and no defects in the fatigue initiation regions were observed for both the alloys. In alloy B, rough and coarse cracks appeared in the fatigue propagation region. The alloy B’s coarse fatigue cracks were also similar to the forms and sizes of PAG. This finding supported the presumption that the MA phases created in PAG facilitated fatigue fracture.

## Figures and Tables

**Table 1:** Chemical compositions of alloys A & B used in this study (in wt. %).

Alloy	Ni	Mn	Cr	C	Si	Mo	Al	V	Ti	B	P	S	Fe
A alloy	3.05	0.96	0.49	0.27	0.26	0.29	0.03	0.03	0.03	0.002	0.001	0.001	Bal.
B alloy	3.00	0.95	0.48	0.28	0.28	0.29	0.03	0.03	0.03	0.002	0.003	0.001	Bal.

**Table 2:** Representative tensile properties of alloys A and B.

Alloys	Y.S. (MPa)	U.T.S. (MPa)	Total El. (%)
Alloy A	1523 (±17)	2120 (±21)	0.042 (±0.002)
Alloy B	1373 (±15)	1645 (±19)	0.043 (±0.002)
